# Correlating biodegradation kinetics of 2,3,7,8-tetrachlorodibenzo-p-dioxin to the dynamics of microbial communities originating from soil in Vietnam contaminated with herbicides and dioxins

**DOI:** 10.3389/fmicb.2022.923432

**Published:** 2022-08-11

**Authors:** Thi Lan Anh Nguyen, Ha Thi Cam Dang, Ton That Huu Dat, Bernd W. Brandt, Wilfred F. M. Röling, Abraham Brouwer, Rob J. M. van Spanning

**Affiliations:** ^1^Department of Molecular Cell Biology, Vrije Universiteit, Amsterdam, Netherlands; ^2^Institute of Biotechnology, Vietnam Academy of Science and Technology, Hanoi, Vietnam; ^3^Mientrung Institute for Scientific Research, Vietnam Academy of Science and Technology, Thua Thien Hue, Vietnam; ^4^Department of Preventive Dentistry, Academic Centre for Dentistry Amsterdam, University of Amsterdam and Vrije Universiteit Amsterdam, Amsterdam, Netherlands; ^5^BioDetection Systems, Amsterdam, Netherlands; ^6^Department of Ecological Science, Vrije Universiteit, Amsterdam, Netherlands

**Keywords:** 2,3,7,8-TCDD, dioxin, biodegradation, Illumina MiSeq sequencing, bacterial community profiling

## Abstract

We studied the succession of bacterial communities during the biodegradation of 2,3,7,8-tetrachlorodibenzo-p-dioxin (2,3,7,8-TCDD). The communities originated from a mesocosm with soil from Bien Hoa airbase in Vietnam heavily contaminated with herbicides and dioxins. They were grown in defined media with different carbon and Gibbs energy sources and 2,3,7,8-TCDD. Cultures with dimethyl sulfoxide (DMSO) as the sole carbon and energy source degraded about 95% of 2,3,7,8-TCDD within 60 days of cultivation. Those with an additional 1 mM of vanillin did that in roughly 90 days. Further 16S rRNA gene amplicon sequencing showed that the increase in relative abundance of members belonging to the genera *Bordetella*, *Sphingomonas*, *Proteiniphilum*, and *Rhizobium* correlated to increased biodegradation of 2,3,7,8-TCDD in these cultures. A higher concentration of vanillin slowed down the biodegradation rate. Addition of alternative carbon and Gibbs energy sources, such as amino acids, sodium lactate and sodium acetate, even stopped the degradation of 2,3,7,8-TCDD completely. Bacteria from the genera *Bordetella*, *Achromobacter*, *Sphingomonas* and *Pseudomonas* dominated most of the cultures, but the microbial profiles also significantly differed between cultures as judged by non-metric multidimensional scaling (NMDS) analyses. Our study indicates that 2,3,7,8-TCDD degradation may be stimulated by bacterial communities preadapted to a certain degree of starvation with respect to the carbon and energy source. It also reveals the succession and abundance of defined bacterial genera in the degradation process.

## Introduction

Polychlorinated dibenzo-p-dioxins (PCDDs) and polychlorinated dibenzofurans (PCDFs), which are commonly named dioxins, are highly toxic, recalcitrant chlorinated compounds and belong to the top of prioritized environmental pollutants in the world (Häggblom and Bossert, 2003; Van den Berg et al., 2006). This group of compounds contains 135 different congeners of PCDFs and 75 different congeners of PCDDs. The congeners with chlorine atoms at the 2,3,7,8 positions have been identified as extremely cytotoxic, while 2,3,7,8-TCDD was declared as the most toxic and carcinogenic compound by the World Health Organization (WHO; Van Den Berg et al., 1998). Dioxins have been detected in dietary food chains such as meat, fish, and egg. They pose serious risks to human health due to their toxicity, mutagenicity, and carcinogenicity (Van den Berg et al., 2006; Squadrone et al., 2015). Dioxins can be formed under natural conditions such as volcanic eruptions and during forest fires (Hoekstra et al., 1999). Furthermore, generation of dioxins also occurs during combustion processes of domestic and industrial waste, bleaching of pulp and metal processing, and during synthesis of chlorinated chemicals (Chang, 2008; Klees et al., 2015; Tue et al., 2016). The most widely used chlorophenoxy herbicides contained a mixture of the n-butyl esters of 2,4-dichlorophenoxyacetic acid (2,4-D) and 2,4,5-trichlorophenoxyacetic acid (2,4,5-T). Such mixtures were used in the Vietnamese war from 1961 to 1971 by the United States military to defoliate trees for strategic reasons. During synthesis of 2,4,5-T at that time, the compound 2,3,7,8-TCDD was formed as a by-product (Westing, 1984; Stellman et al., 2003). Drums containing the herbicides and 2,3,7,8-TCDD were stored at former military bases in Bien Hoa, Phu Cat and Da Nang. During handling and the use of these herbicides, many leakages occurred, such as storage leakages, or leakages from washing and loading of the Ranch Hand aircrafts. Until 2009, Bien Hoa airbase was a hotspot with a high contamination of herbicides and dioxins (Dwernychuk et al., 2002; Mai et al., 2007).

The detoxification and degradation of dioxins from polluted environments is currently one of the most interesting and challenging issues in environmental remediation technologies. Physicochemical approaches, such as photodegradation, thermal remediation, supercritical hydrolysis and dechlorination with a metal catalyst have all been applied in dioxin remediation. Nevertheless, these technologies were not very effective for large areas because they are very costly and have a severe negative impact on the environmental ecology because they end up introducing new chemical wastes into the environment (Kulkarni et al., 2008; Lin et al., 2015; Liu et al., 2015; Palanisami et al., 2015). Bioremediation is usually considered as a much more cost-effective alternative with less impact on the environment compared to both chemical and physical methods for remediation. Bioremediation approaches, such as augmentation technologies, use indigenous microbial consortia for the biodegradation of toxic and persistent compounds from the polluted environment (Chang, 2008; Jeon et al., 2016). A number of studies revealed the importance of some bacterial strains in biodegradation of dioxins, such as *Sphingomonas wittichii* RW1 (Hong et al., 2002), *Nocardioides aromaticivorans* (Kubota et al., 2005), *Rhodococcus* sp. strain HA01 (Aly et al., 2008), *Rhodococcus* sp. strain p52 (Peng et al., 2013), *Pseudomonas* sp. strain CA10 (Sato et al., 1997), *Pseudomonas mendocina* strain NSYSU (Lin et al., 2014) and *Agrobacterium* sp. PH-08 (Le et al., 2014). *Rhodococcus* sp. strain HA01 and *Pseudomonas* sp. strain CA10 seemed to be particularly interesting since they have genes encoding enzymes participating in the biodegradation of dibenzofuran (DF) and dibenzo-p-dioxin and converted these contaminants into less toxic metabolites (Sato et al., 1997; Aly et al., 2008). However, most studies have mainly focused on the aerobic biodegradation of less toxic dioxin congeners and information on the toxic 2,3,7,8-TCDD degradation pathways is scarce (Chen et al., 2014).

Since only about 1% of the total number of bacteria present in the environment can be cultivated on laboratory media (Ferrari et al., 2005; Vartoukian et al., 2010), high-throughput molecular techniques such as Illumina MiSeq amplicon sequencing are used nowadays to gain a better understanding of the composition and dynamics of complex microbial communities (Kowalczyk et al., 2015; Gutleben et al., 2018; Liu et al., 2019; Lu et al., 2019; Poursat et al., 2019).

In this study, we aimed at correlating biodegradation kinetics of 2,3,7,8-TCDD to the dynamics of microbial communities during enrichment cultivation in order to get a better understanding of the key bacteria participating in 2,3,7,8-TCDD biodegradation under laboratory conditions. Our specific approach was to culture bacterial communities enriched from polluted soil from Bien Hoa airbase on mineral salt medium with different aromatic carbon and energy sources such as vanillin and aromatic amino acids with the rationale that such aromatic compounds would induce the recruitment of mono-and dioxygenases ultimately to enhance the rate of dioxin degradation. We also used sodium salicylate because it is an intermediate compound in the pathway of dioxin degradation. We, therefore, hypothesized that this compound could be used to promote cometabolism of 2,3,7,8-TCDD. We then monitored the degradation of 2,3,7,8-TCDD and the resulting changes in bacterial community structures over time and performed correlation analyses. As a result, we obtained a more fundamental understanding of the conditions for biodegradation of 2,3,7,8-TCDD as well as of bacterial genera that became abundant during biodegradation of 2,3,7,8-TCDD in these enrichment cultures.

## Materials and methods

### Soil samples and culturing conditions

Herbicide and dioxin polluted soil for the enrichment experiments was collected from Bien Hoa airbase (10°58′14.3″ N 106°48′19.3″ E), Dong Nai province, Vietnam. For that, five holes were dug 2.5–3 m deep from the surface by an excavator. Samples were taken at about 20 to 25 different positions of the piles of soil from each hole and well mixed on-site, transferred to Hanoi, and then divided over 6 containers, each with a volume of 200 L and filled with 140 kg of soil. The containers were periodically supplied with water to keep the moisture between 12 and 20%. One container, called T1, was left untreated for 38 months. The other five were biotreated for the same period each with a different design. We took soil samples at the start of bioremediation and after 6, 21, and 38 months for further studies (Nguyen et al., 2021b). Untreated soil T1 was sampled after 21 months for the culturing experiments described in this experiment. 2,3,7,8-TCDD (10 μg/ml in toluene) was purchased from Sigma-Aldrich Co., United States and was prepared as a stock solution of 1 μg/ml in dimethyl sulfoxide (DMSO). It was added in mineral salt medium for degradation assays at final concentrations of 0.5 ng 2,3,7,8-TCDD /ml in 7 mM DMSO. Five grams of soil from the mesocosm was inoculated in 250 ml Erlenmeyers flasks filled with 45 ml BS medium per culture containing KH_2_PO_4_ 0.5 g/l, (NH_4_)_2_SO_4_ 0.25 g/l, MgSO_4_ 0.2 g/l, CaCl_2_ 0.5 g/l and NaNO_3_ 0.4 g/l, pH 7.0 (van der Zaan et al., 2012). All the cultures contained 7 mM DMSO. One of these flasks was left without additional carbon and energy sources, the others contained either 1 or 5 mM vanillin, or 3 mM aromatic amino acids (1 mM tryptophan, 1 mM tyrosine and 1 mM phenylalanine), or 5 mM sodium salicylate or a mixture of 12 mM sodium acetate and 9 mM sodium lactate. These cultures were incubated in the dark on a rotary shaker at 30°C and 200 rpm. After 10 days, 10 ml of these cultures (1^st^ enrichments) were then used to inoculate 90 ml of fresh BS medium with the same carbon and energy sources described above and further incubated by shaking at 200 rpm and 30°C. After another 10 days, 10 ml of these cultures (2^nd^ enrichments) were then transferred into 90 ml fresh BS medium with the carbon and energy sources described above. The 3^rd^ enrichments steps were carried out the same way as was done for the 2^nd^ enrichments. After 10 days, 10 ml of the 3^rd^ enrichments were then transferred into 90 ml fresh BS medium with the carbon and energy sources described above and with the addition of 2,3,7,8-TCDD at final concentrations of 0.5 ng 2,3,7,8-TCDD /ml in 7 mM DMSO and further incubated by shaking at 200 rpm and 30°C for 115 days. At several time points during the incubation time, aliquots of the incubation medium were collected for analyses of 2,3,7,8-TCDD activity by the DR CALUX^®^ (Dioxin Responsive-Chemical activated luciferase gene expression) bioassay (as described later) and bacterial colony forming units (CFUs) and community profiling. The control flask containing 2,3,7,8-TCDD in DMSO for non-biological degradation of 2,3,7,8-TCDD contained 10 ml of bacterial cells from the 3^rd^ enrichments that were sterilized by heating them at 121°C during 15 min prior to incubation. The number of cultivation days for each experiment is mentioned in the results section. All experiments were carried out in duplicate. A diagram with the setup of the enrichment experiments described in this study is shown in Supplementary Figure S1.

### Rationale for random sampling

We aimed at studying the succession in time of bacterial cultures growing on 2,3,7,8-TCDD and to correlate bacterial community structures and their changes to their corresponding degradation capacities. Therefore, we investigated if 2,3,7,8-TCDD was homogenously distributed during random sampling. For that, 5 ml of homogenate from flasks containing 50 ml of BS medium and 0.5 ng 2,3,7,8-TCDD /ml was taken for dioxin extraction in triplicate.

### Bacterial community profiling using Illumina sequencing of the 16S rRNA gene amplicon

Enrichment cultures were sampled at 0, 10, 28, 45, 60, 90 and 115 days of incubation for DNA extraction. Genomic DNA of cultures was extracted using the MoBio PowerSoil^®^ DNA Isolation kit (Carlsbad, CA, United States). The concentration of the DNA was measured by the Qubit dsDNA HS Assay kit (ThermoFischer Scientific, cat. no. Q32851). The primers target the V3-V4 region of bacterial 16S rRNA genes and will yield a ~ 550 bp fragment during PCR. Each of the 24 primers (8 forward primers; 16 reverse primers) contains a specific index sequence can be found in the supplementary material (Supplementary Table S1). These amplicon primers contained Illumina adapters and an 8-nucleotide index barcode sequence (Kozich et al., 2013). The PCR system and amplification conditions were similar to those described by Mao et al. (2015). The composition of a PCR reaction was as follows: 5 μm primers, 5x Phusion HF Green Buffer, 10 mM dNTP mix, 2 U/μL Phusion II HS HF Polymerase, and 1.5 ng DNA template. The PCR program used for amplification used for amplicon sequencing can be found in the supplementary material (Supplementary Table S2). The PCR products were purified with Agencourt AMPure XP magnetic beads (Beckman Coulter, Brea, CA, United States) on a BILATEST magnetic separator plate (Sigma Aldrich, cat. no.: Z662429-1EA). Subsequently, purified PCR products were visualized using gel electrophoresis and quantified with a Qubit dsDNA HS Assay Kit on a Qubit 2.0 fluorometer (Thermo Fisher Scientific). The libraries were sequenced on the Illumina MiSeq platform (Illumina Inc.; San Diego, CA, United States) with 150 cycles for forward and reverse reads.

### Sequence processing and analyses

The sequencing data reads were processed as described previously (Persoon et al., 2017). In short, the reads were merged (Edgar and Flyvbjerg, 2015), filtered, and clustered into operational taxonomic units (OTUs) in line with the UPARSE method using USEARCH version 8.0.1623 (Edgar, 2013). Sequences to be mapped to the cluster centroids were filtered on a maximum error rate of 0.005, while no ambiguous bases were allowed. QIIME version 1.8.0 (Caporaso et al., 2010) was used to select the most abundant sequence of each OTU, the taxonomy of which was assigned using the RDP classifier (Wang et al., 2007) with a minimum confidence of 0.8 and the 97% representative sequence set V3-V4 region only, made non-redundant (*cf.* Koopman et al., 2016) based on the SILVA rRNA database v132 (Quast et al., 2013). For the taxonomic summaries of the bacterial communities at phylum and genus levels, the counts in the OTU table were converted to relative abundances. Analyses of (i) alpha diversity indices (species richness and Shannon index), of (ii) NMDS (PERMANOVA and dispersion) and of (iii) log2-fold changes in abundance of OTUs are described elsewhere (Nguyen et al., 2021b). Before the alpha-and beta-diversity analyses (including NMDS), the OTU table was standardized using the *decostand* function (method = “hellinger”; Steinert et al., 2017, 2019; Helber et al., 2019) of the R package vegan v.2.5-6 (Oksanen et al., 2016) in R v3.3.1 (R Core Team, 2016). The alpha diversity indices, species richness (S, number of OTUs/sample), and Shannon index (H) were calculated in R using vegan. The difference in alpha diversity indices of bacterial communities from cultures in BS medium during growth on different carbon and energy sources was analysed with the Kruskal–Wallis rank-sum test using the function *kruskal.test* followed by Dunn’s Kruskal-Wallis multiple comparisons test using the function *dunnTest* within the package FSA v.0.8.30 (Ogle, 2017) with the Benjamini-Hochberg (BH) value of *p* correction for multiple testing (Benjamini and Hochberg, 1995). The non-metric multidimensional scaling (NMDS) plot was created *via* the function *metaMDS* (Bray–Curtis distances). Confidence ellipses in NMDS plots were shown at a 0.95 level using the function *ordiellipse* (kind “sd”) of the vegan package. Multivariate analysis based on Bray–Curtis dissimilarities (1,000 permutations) of bacterial communities based on cultures in BS medium during growth on different carbon and energy sources, followed by pairwise comparisons of different carbon and energy sources were performed using the functions *betadisper*, *permutest* (dispersion), *adonis* (PERMANOVA) from vegan, and the function *pairwise*.*perm*.*manova* of the package RVAideMemoire v.0.9–77 (Hervé, 2020) with the BH value of *p* correction for multiple testing.

The OTU differential abundance (log_2_-fold change in abundance of each OTU) between two groups (i.e., between two culture periods of cultures in BS medium during growth on different carbon and energy sources) was calculated using the R package phyloseq v.1.32.0 (McMurdie and Holmes, 2013) and ‘DESeq2’ v1.28.1 (Love et al., 2014). For this analysis, the original OTU table was normalized internally with DESeq2. The value of ps were adjusted with the BH correction method and an OTU was considered as differentially abundant if its mean proportion was significantly different between sample classes (value of *p* < 0.05). Only OTUs with a relative abundance of more than 2% in total were selected for these analyses.

### Analysis of 2,3,7,8-TCDD by the DR-CALUX^®^ bioassay

2,3,7,8-TCDD was quantified using the DR-CALUX^®^ bioassay (Dao et al., 2019). This bioassay is based on the activation of a mammalian aryl hydrocarbon receptor (AhR) by ligands such as dioxins or dioxin-like compounds, and is optimized for detection of 2,3,7,8-TCDD (Besselink et al., 2004; Hoogenboom et al., 2006; van Vugt-Lussenburg et al., 2013; Dao et al., 2019). 2,3,7,8-TCDD from 5 ml of culture samples was extracted using a mixture of 5 ml of isopropanol and 10 ml of hexane (1:2) and was shaken at 100 rpm for 30 min. Then, the hexane phase containing 2,3,7,8-TCDD was transferred to a glass vial. The residual layer was re-extracted twice with hexane. The hexane in the vial was evaporated to approximately 2 ml. Subsequently, the hexane fraction was cleaned on a column containing 10 g acid silica using 20 and 33% H_2_SO_4_ at a 1:1 ratio as described earlier. The cleaned extract was further concentrated to near dryness under a gentle stream of nitrogen. Finally, the extract was transferred to a dark clean vial after which 250 μl of DMSO was added for the DR-CALUX^®^ bioassay.

### Data accessibility

The raw Illumina sequencing data were deposited at the National Center for Biotechnology Information (NCBI) Sequence Read Archive (SRA) under the Bioproject number PRJNA682511.

## Results

### Rationale for random sampling

The results showed no significant difference between three replicates of random sampling for 2,3,7,8-TCDD extraction while the samples indeed contained a concentration of 2,3,7,8-TCDD close to what was calculated (Supplementary Figure S2). Therefore, we used random sampling for 2,3,7,8-TCDD extraction in this study.

### Biodegradation of 2,3,7,8-TCDD by bacterial communities enriched from soil polluted with herbicides and 2,3,7,8-TCDD

The soils had an average dioxin toxicity of 21.605 ngTEQ/kg at the start of bioremediation (Supplementary Table S4). Concentrations of 2,4-D and 2,4,5-T at 21 months of bioremediation were 891 mg/kg soil and 1,375 mg/kg soil, respectively. The carbon content in the soil was 0.55% and from that number we calculated that the concentrations of remaining carbon in the subsequent 1^st^, 2^nd^, and 3^rd^ enrichments were 0.6, 0.06 and 0.006 μg/ml, respectively. Most likely, that carbon may be used as carbon and energy source as well, but the concentration is much lower than that of the added sources. For that reason, its metabolism would hardly affect the community composition, especially in the 3^rd^ enrichment.

**Figure 1:**
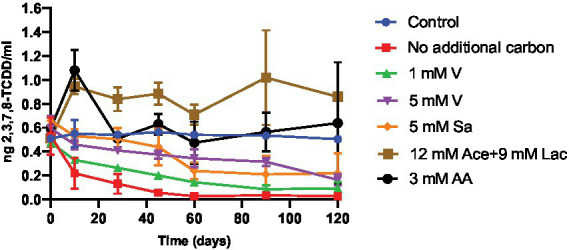
Degradation of 2,3,7,8-TCDD, as measured by the DR^®^ CALUX bioassay, over time in cultures containing different carbon and energy sources. 2,3,7,8-TCDD was dissolved in DMSO and added to culture flasks to a final concentration of 0.5 ng/ml. The DMSO concentration was 7 mM in all cultures. The error bar at each data point represents the standard deviation of two independent experiments. Blue circles, control incubations with dead cells obtained after heat inactivation at 121°C for 15 min (control); red squares, no additional carbon and energy sources; green triangles, 1 mM vanillin (1 mM V); purple triangles, 5 mM vanillin (5 mM V); orange diamonds, 5 mM sodium salicylate (5 mM Sa); brown squares, 12 mM sodium acetate and 9 mM sodium lactate (12 mM Ace +9 mM Lac); black circles, 3 mM amino acid including 1 mM tryptophan, 1 mM tyrosine and 1 mM phenylalanine (3 mM AA).

2,3,7,8-TCDD degradation and the number of CFUs over time as an indicator for biomass formation were determined in samples collected at several different time points from enrichment cultures with BS medium during 115 days of cultivation (Figure 1; Supplementary Figure S3). Different carbon sources were added in the enrichment cultures to study their effects on 2,3,7,8-TCDD breakdown. The 2,3,7,8-TCDD concentration by the DR-CALUX^®^ bioassay, and bacterial growth (number of CFUs) in the culture flasks was determined at various incubation time points. The cultures in BS medium with only DMSO showed a maximum increase of CFUs after 28 days and a degradation of 94% of 2,3,7,8-TCDD within 60 days of incubation. This was the highest degradation percentage observed as compared to the other cultures that all had DMSO as well but with additional carbon and energy sources. The cultures in medium supplemented with 1 mM vanillin showed an increase in the number of CFUs, reaching a maximum after 10 days of growth. These cultures degraded 73% of 2,3,7,8-TCDD during 60 days of cultivation. The cultures in BS medium with 5 mM vanillin showed a small increase in the number of CFUs after 10 days of cultivation, which then reduced after 90 days, most likely because of bacterial death. These cultures degraded only 36% of the supplied 2,3,7,8-TCDD during 60 days of incubation. Cultures on BS medium with 5 mM sodium salicylate showed a maximum number of CFUs after 10 days of growth and removed 56% of 2,3,7,8-TCDD during 60 days of cultivation. Cultures containing a mixture of aromatic amino acids or with a mixture of sodium acetate and sodium lactate also showed an increase in the number of CFUs, but neither of these two cultures degraded 2,3,7,8-TCDD over time. We noticed a slight increase in the toxicity of extracted sample in the culture with the amino acids and a subsequent decrease again to the original level within the first 30 days of cultivation, and we suggest that this may be an unwanted effect of (one of) the aromatic amino acids on the DR-CALUX^®^ bioassay that disappears again after consumption of such amino acid(s) by the bacterial community. In contrast, the addition of a mixture of 12 mM sodium acetate and 9 mM sodium lactate as additional carbon and energy sources to the culture medium showed an initial increase of the toxicity of extracted sample that maintained stable throughout the whole experiment.

### Community dynamics of 2,3,7,8-TCDD degrading cultures

DNA of the cultures that were sampled after different time points of incubation was extracted and sequenced using 16S rRNA gene amplicon sequencing. From the 4,095,916 read pairs produced, 3,141,045 (77%) sequences were mapped to 134 OTUs during data processing. From the sequences remaining after quality filtering (no ambiguous bases, max EE rate 0.005, *cf.* Koopman et al., 2016), 98.4% mapped to the OTUs. Good’s coverage values were calculated based on subsampling size of the sample with the fewest sequences (n = 12,115 reads). The good’s coverage values and the number of reads ranged from 99.88 to 100% and 12,115 to 111,300, respectively (Supplementary Table S3). The obtained Operational Taxonomic Unit (OTU) tables were used for alpha-diversity analysis. The results show that both the Chao1 richness and Shannon indices of cultures supplemented with the different carbon sources were significantly different from each other (Supplementary Figure S4).

**Figure 2:**
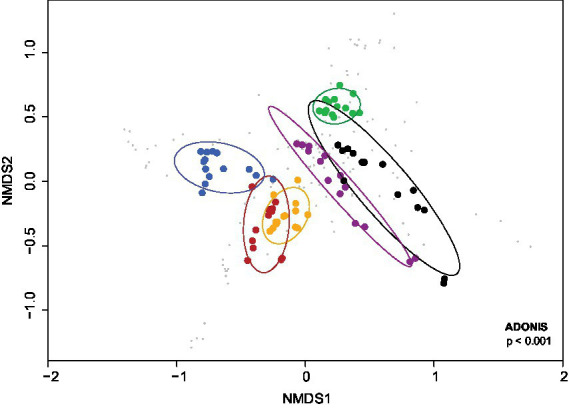
NMDS ordination of the Bray–Curtis dissimilarity of bacterial communities from cultures in BS medium during growth on different carbon and energy sources in addition to 7 mM DMSO. Orange, 3 mM aromatic amino acids; green, no additional carbon and energy source; red, sodium acetate and sodium lactate; blue, 5 mM sodium salicylate; purple, 1 mM vanillin; black, 5 mM vanillin. The cultures were sampled at 0, 10, 28, 45, 60, 90 and 115 days of incubation with duplicates. Confidence ellipses level at 0.95. values of *p* are at the lower right corner of the plot.

The NMDS analysis showed a significantly different pattern of bacterial communities from these cultures in BS medium supplemented with different carbon sources (dispersion value of *p* < 0.001; Figure 2). Apparently, the carbon and energy sources added to BS medium resulted in changes of the community structure/profile and succession of the bacterial communities during cultivation. In addition, permutational multivariate analysis of variance (PERMANOVA value of *p* <0.001) indicated that the compositions of bacterial communities in cultures with different carbon and energy sources significantly differed.

**Figure 3:**
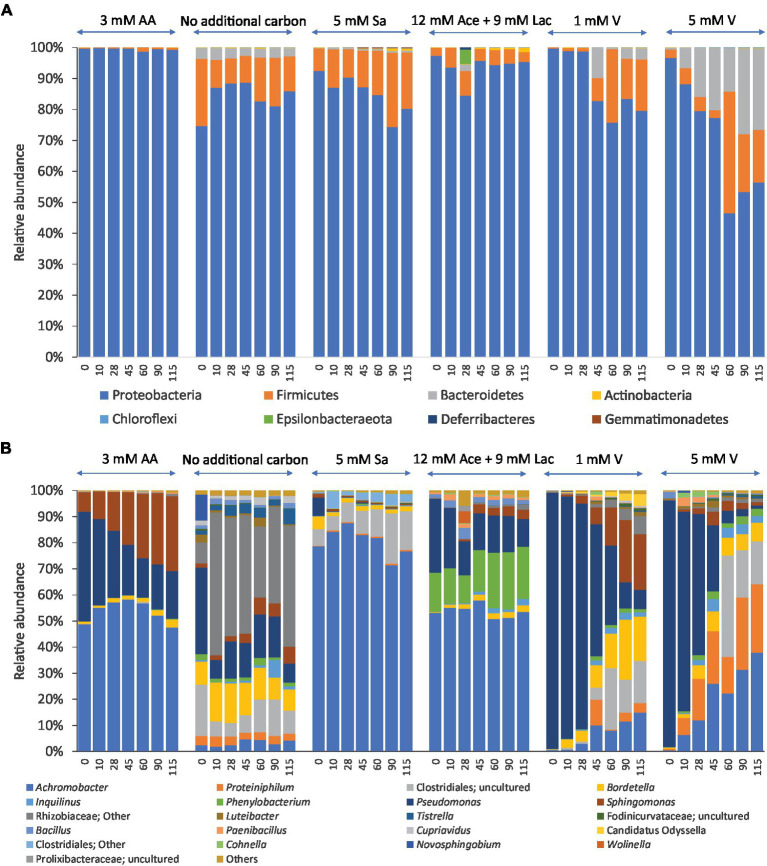
Relative abundance of bacteria at the phylum level **(A)** and genus (or higher) level **(B)** of enrichments with different carbon and energy sources and 2,3,7,8-TCDD in 7 mM DMSO during 115 days of incubation. The sources were 3 mM aromatic amino acids (3 mM AA), no additional carbon and energy sources, 5 mM sodium salicylate (5 mM Sa), a mixture of 12 mM sodium acetate and 9 mM sodium lactate (12 mM Ace+9 mM Lac), 1 mM vanillin (1 mM V), 5 mM vanillin (5 mM V). The data shown are the averages of two replicates. Genera with relative abundances less than 2% are grouped as ‘Others’.

Analysis of the relative abundances showed a total of 8 phyla found in all samples of enriched communities. Three of them were abundant in enrichments with different carbon sources at all-time points, namely Proteobacteria (44.1–99.9%), Firmicutes (2.1–47.7%) and Bacteroidetes (2.1–29.9%). Proteobacteria was the most dominant phylum in all these cultures, most pronounced in cultures with amino acids as carbon and energy sources where its members represented over 90% of the bacterial communities at all-time points of incubation (Figure 3A). A lower abundance in Proteobacteria, specifically of the Gammaproteobacteria, was accompanied by a higher abundance of the Firmicutes and Bacteroidetes in cultures with 5 mM vanillin as carbon source after 10 days of incubation. Other phyla such as Actinobacteria, Gemmatimonadetes and Deferribacteres had less than 2% relative abundance across all consortia (Figure 3A).

The relative abundances of OTUs that belong to the first 21 most abundant genera are shown in Figure 3B. Members of the genus *Achromobacter* were found at all-time points of incubation regardless of the type of carbon and energy source that was added to these cultures. Three genera, *Sphingomonas* (7–30%), *Pseudomonas* (14–46%) and *Achromobacter* (45–60%) were dominant in the cultures with the mixture of amino acids as carbon and energy sources over time. Moreover, members of the genus *Achromobacter* had relative abundances between 51 and 88% in the cultures with sodium salicylate and a mixture of sodium acetate and sodium lactate as carbon and energy sources. Members of the genus *Pseudomonas* dominated in all cultures at the start of the experiment with relative abundances between 20 and 99%, but these decreased over time. On the other hand, members of the order Clostridiales and genera *Bordetella* and *Sphingomonas* had a low relative abundance at the start of the experiment and became more abundant during the enrichment in cultures with no additional carbon and energy sources other than DMSO, and in the cultures with additional 1 mM and 5 mM vanillin. Members of the Rhizobiaceae and *Proteiniphilum* were dominant in the cultures with only DMSO as the carbon source at all different time points.

**Figure 4:**
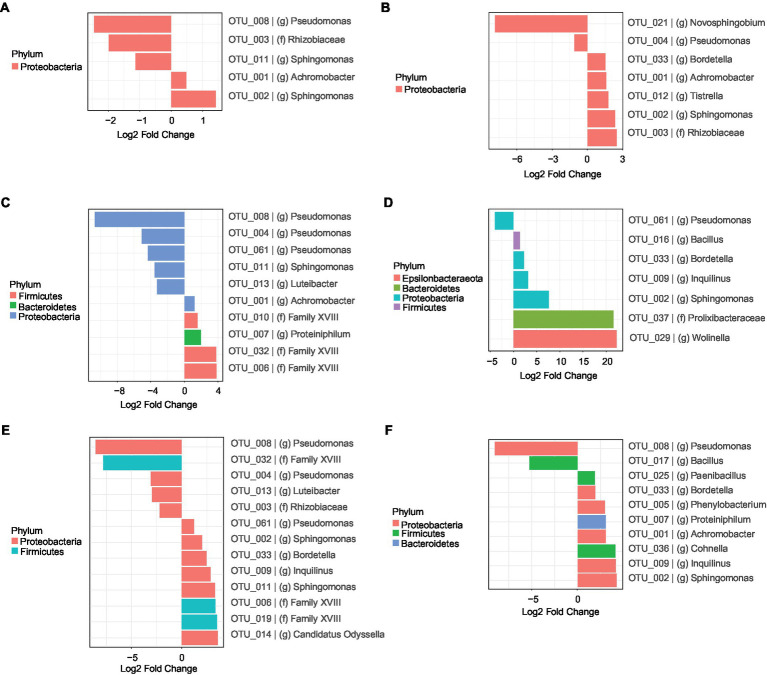
Differential analysis (Log_2_-fold changes) representing each OTUs whose relative abundance changed significantly (*p* < 0.05) after 28 days of incubation in comparison with the original inoculum (0 day) from cultures in BS medium during growth on different carbon and energy sources in addition to 7 mM DMSO. **(A)**, a mixture of 3 aromatic amino acids including 1 mM tryptophan, 1 mM tyrosine and 1 mM phenylalanine; **(B)**, no additional carbon and energy sources; **(C)**, 5 mM sodium salicylate; **(D)**, a mixture of 12 mM sodium acetate and 9 mM sodium lactate; **(E)**, 1 mM vanillin; **(F)**, 5 mM vanillin. The bars of the OTUs are coloured according to the colour scale of the phyla that they belong to.

To get a better understanding of the succession of the bacterial communities and a possible role of species in the degradation of 2,3,7,8-TCDD, we determined Log_2_-fold changes of OTUs in selected time intervals. We first analyzed the succession of the bacterial communities during the first 28 days of the enrichment (Figure 4). In BS medium with aromatic amino acids as additional carbon and energy sources, bacteria from the genera *Sphingomonas* (OTU-002) and *Achromobacter* (OTU-001) were more abundant after 28 days of incubation in comparison with the original bacterial consortia. The relative abundance of another 3 OTUs from the phylum Proteobacteria, including the genera *Pseudomonas* (OTU-008), *Sphingomonas* (OTU-011) and a member of the family Rhizobiaceae, decreased after 28 days of the experiment (Figure 4A). Cultures with only DMSO as added carbon and energy sources revealed that the relative abundances of 5 OTUs, from genera *Bordetella* (OTU-033)*, Achromobacter* (OTU-001)*, Tistrella, Sphingomonas* (OTU-002) and a member of the family Rhizobiaceae, significantly increased while those from another 2 genera, *Pseudomonas* (OTU-004) and *Novosphingobium,* decreased after 28 days of cultivation compared to the start of the experiment (Figure 4B).

Cultures with sodium salicylate as added carbon and energy source showed an increase in relative abundances of the genera *Achromobacter* (OTU-001) and *Proteiniphilum* and members of the phylum Firmicutes. The relative abundances of the genera *Pseudomonas* (OTU-004, OTU-008 and OTU-061), *Luteibacter* and *Sphingomonas* (OTU-011) decreased after 28 days of cultivation in comparison with those of the original communities (Figure 4C). Cultures supplemented with a mixture of sodium lactate and sodium acetate showed a total of 6 OTUs, belong to the genera *Bacillus*, *Bordetella* (OTU-033), *Sphingomonas* (OTU-002), *Inquilinus*, *Wolinella* and a member of the family Prolixibacteraceae whose relative abundance significantly increased after 28 days of incubation compared to the start of the experiment. In contrast, the relative abundances of OTUs belonging to the genus *Pseudomonas* (OTU-061) decreased in the same period (Figure 4D).

Cultures with additional 1 mM vanillin showed a significant increase of OTUs from the genera such as *Pseudomonas* (OTU-061), *Bordetella* (OTU-033), *Sphingomonas* (OTU-002), *Inquilinus*, *Candidatus Odyssella* and genera from the phylum Firmicutes and a decrease of OTUs from genera *Pseudomonas* (OTU-004 and OTU-008), *Luteibacter*, genera from family Rhizobiaceae and genera from the phylum Firmicutes (Figure 4E). Those with 5 mM vanillin added showed increased relative abundances of the genera *Paenibacillus*, *Cohnella*, *Sphingomonas* (OTU-002)*, Achromobacter* (OTU-001), *Bordetella* (OTU-033), *Phenylobacterium*, *Proteiniphilum* and *Inquilinus*. In contrast, the relative contribution of members of the genera *Bacillus* and *Pseudomonas* (OTU-008) decreased after 28 days of the experiment (Figure 4F). Cultures incubated longer than 28 days showed additional changes in the composition of the microbial communities, but these changes are most likely not induced by the carbon and energy sources added at the start of the experiment as these are most likely fully consumed during the first 28 days of cultivation. (Supplementary Figure S5).

## Discussion

We studied the dynamics of bacterial communities originating from soil contaminated with herbicides and 2,3,7,8-TCDD and correlated some of their characteristics to the breakdown of 2,3,7,8-TCDD. Cultures that showed the highest rates of 2,3,7,8-TCDD breakdown had only DMSO present as available carbon and energy source. The culture with an additional 1 mM vanillin showed a reduction of degradation by 22% compared to the culture with only DMSO. These two cultures had in common that they showed a higher abundance of the genera *Bordetella* (OTU-033) and *Sphingomonas* (OTU-002) during the first 28 days of cultivation. It should be noted here that virtually nothing is known about metabolism of DMSO in these organisms. On the other hand, the presence of enzymes for degradation of various aromatic compounds in *Bordetella* species is well documented (Gross et al., 2008). Importantly, the increase in CFUs/ml of *Bordetella* and *Sphingomonas*, as an indicator for biomass formation, occurred within the first 28 days in all cultures. It is, therefore, reasonable to assume that the carbon and Gibbs energy sources that were added to these cultures were metabolized for biomass formation well within that time span. We hypothesize that maintenance and growth of the species present in these cultures after 28 days of cultivation is supported by feeding on necromass and of remaining metabolites. In that time span, one or more of the remaining species apparently continued with the degradation of 2,3,7,8-TCDD albeit at rates depending on the type of carbon and energy source that had been added. Importantly, the concentration of 2,3,7,8-TCDD added (0.5 ng /ml) was so low that its consumption and subsequent metabolism could hardly have contributed to the biomass produced by the metabolism of the additional carbon and energy sources that were added at the start of the experiment because their concentration (in the millimolar range) was much higher. This means that the development of the communities in all cultures is not the result of 2,3,7,8-TCDD degradation, but almost completely dependent on these added carbon and energy sources. Previous studies also reported that the type of carbon source added to the growth medium has a profound effect on the composition of bacterial communities and the abundances of species therein. Carbon levels also affect the bacterial community composition, i.e., species favoured by an excess of carbon sources may grow faster or more efficiently under these conditions, causing a change in bacterial community composition as well as in increased abundances of participating genera (Wawrik et al., 2005; Flynn et al., 2017).

We further showed that members of the genera *Bordetella* and *Sphingomonas* had a low relative abundance at the start of the experiment and became more abundant with time, both in cultures without additional carbon and energy sources other than DMSO and in the cultures with an additional 1 mM of vanillin. Although there is no direct evidence for the involvement of *Bordetella* and *Sphingomonas* in the degradation of 2,3,7,8-TCDD, the fact that they are relatively dominant in these 2 types of culture makes it a likely assumption. It has been shown earlier that species from the genera *Bordetella* and *Sphingomonas* have the genes encoding the enzymes degrading 2,4-D and 2,4,5-T, and indeed use that potential to degrade these herbicides (Nguyen et al., 2021a,b). We suggest that the presence of the herbicides 2,4-D and 2,4,5-T in the soil has contributed to the bioremediation results. Because these compounds are related aromatic compounds, they may well stimulate the indigenous microorganisms to metabolize dioxins in soil. Many other studies reported that members of the genus *Sphingomonas* were able to degrade several types of dioxins such as DD, DF, 2,7-dichlorodibenzo-p-dioxin, 1,2,3-trichlorodibenzo-p-dioxin, 1,2,3,4-tetrachlorodibenzo-p-dioxin, and 1,2,3,4,7,8-hexachlorodibenzo-p-dioxin (Wittich et al., 1992; Wilkes et al., 1996; Megharaj et al., 1997; Halden et al., 1999; Ishiguro et al., 2000; Hong et al., 2002, 2004; Nam et al., 2005, 2006) and for that reason they are regarded as potential candidates for bioremediation of dioxins from contaminated environments (Halden et al., 1999; Hiraishi, 2003; Nam et al., 2005). The other two cultures able to degrade 2,3,7,8-TCDD, albeit at lower rates than the former ones with only DMSO or with an additional 1 mM vanillin, contained an additional 5 mM vanillin or 5 mM salicylate. Also, in the case of 5 mM vanillin, an increase in relative abundance of genera *Bordetella* and *Sphingomonas* was observed, supportive of the assumption that these two genera are important for carbon metabolism in these cultures, and possibly also for co-metabolism of 2,3,7,8-TCDD.

Some other genera also showed increased abundances during the first 28 days of the culture in the presence of 5 mM vanillin, which were not observed in the former two cultures. One of these is *Achromobacter*, which also became more dominant in the cultures with sodium salicylate, a mixture of amino acids or only DMSO as added carbon source. The cultures unable to degrade 2,3,7,8-TCDD, those with a mixture of amino acids and those with a mixture of lactate and acetate, also showed increases in relative abundance of genera *Bordetella*, *Achromobacter* and *Sphingomonas* during the first 28 days of cultivation, but these strains became much less abundant during prolonged culturing as compared to the cultures with only DMSO and those with additional 1 mM vanillin. In the presence of multiple carbon and energy sources, bacteria can selectively utilize an easily degradable carbon source without expressing the enzymes that catalyze less preferred sources (Stülke and Hillen, 1999; Brückner and Titgemeyer, 2002). Thus, when bacteria utilize carbon sources such as the mixture of amino acids or the mixture of sodium lactate and sodium acetate as preferred carbon and Gibbs energy sources, an inhibition in 2,3,7,8-TCDD degradation may be expected to occur. There seems to be a delicate balance in the required addition of carbon to contaminated soils with different effects on the degradation of the contaminants since it has also been reported that carbon added to contaminated soils with low concentrations of xenobiotics may lead to improved biodegradation of these pollutants (Abdelhafid et al., 2000; Lee et al., 2003; Sarkar et al., 2005; van Herwijnen et al., 2006; Dehghani et al., 2013; Nzila, 2013; Suttinun et al., 2013).

On the other hand, excessive addition of carbon sources in culture media may slow down the rate of the degradation of pollutants (Barriuso et al., 1997; Teng et al., 2010; Khleifat et al., 2015; Lee et al., 2020). Vanillin, which may well be a protonophore as judged from its structure, has been reported to be toxic to many microorganisms at high concentrations because it may indeed disrupt the cytoplasmic membrane potential and increase cell membrane permeability resulting in leakage of cellular components, inhibition of respiration and the dissipation of ion gradients (Fitzgerald et al., 2004; Yang et al., 2021). Indeed, high concentrations of vanillin inhibit microbial growth leading to cell death (Fitzgerald et al., 2004; Oliva et al., 2004; Yang et al., 2021). Accordingly, our cultures with vanillin at a concentration of 5 mM performed worse with respect to 2,3,7,8-TCDD degradation than those with only 1 mM, suggesting that the excess of vanillin had a negative effect on bacterial growth resulting in inhibition of 2,3,7,8-TCDD degradation. This was also suggested by the culture with 5 mM vanillin showing 100 times fewer CFUs over time than the culture with 1 mM vanillin (Supplementary Figure S3).

The increase in the toxicity of extracted sample was observed in the cultures by adding the mixture of sodium acetate and sodium lactate. This apparent increase in the level of toxicity of extracted sample is most likely due to an artefact caused by the presence of sodium acetate, which has recently been indicated as a natural enhancer of AhR mediated gene transcription similar to other short chain fatty acids (SCFAs). Therefore, it may give rise to an artificially higher than expected luminescence readout which when interpolated on the dose–response curve of the used reference standard will give rise to an apparently higher dioxin TEQ value (Jin et al., 2017). The DR-CALUX^®^ method may determine other sulfuric acid stable, AhR active compounds, such as brominated dioxins and furans and some nitrated polycyclic aromatic hydrocarbons (nitro-PAHs) which are not yet regulated by authorities and therefore, the DR-CALUX^®^ method may give rise to somewhat higher levels of dioxins and dioxin-like compounds as compared to the High Resolution Gas Chromatography Mass Spectrometry (HR-GC–MS) method, which only determines 27 congeners of the total number of toxic dioxin-like chemicals, regulated in food and feed safety testing. This means that in some cases the DR-CALUX^®^ method may respond to sufuric acid stable, AhR ligands which are chemically not classified as dioxins, but show a similar toxicological response as dioxins and dioxin-like compounds (Budin et al., 2020).

Taken all together, we showed that the culture with the lowest concentration of added carbon and energy sources, the one with only DMSO, performed best with regard to the degradation of 2,3,7,8-TCDD. All others with additional carbon and energy sources performed less well (the vanillin and salicylate cultures) or were unable to degrade 2,3,7,8-TCDD at all (cultures with the mixture of aromatic amino acids and the mixture of lactate and acetate). We have seen a similar phenomenon in a study on the degradation of the herbicides 2,4-D and 2,4,5-T, in which the best performance was attributed to bacterial communities relatively starved in amended, more easily degradable carbon and energy sources (Nguyen et al., 2021b). Perhaps also in this study, the degraders of xenobiotics are outcompeted by strains that happily grow on the amended sources and consume essential nutrients and vitamins required by the degraders.

## Data availability statement

The datasets presented in this study can be found in online repositories. The names of the repository/repositories and accession number(s) can be found in the article/Supplementary material.

## Author contributions

TLAN designed and performed the research, analysed the data, prepared figures and tables, authored or reviewed drafts of the paper, and approved the final draft. HTCD delivered soil material, reviewed drafts of the paper, and approved the final draft. TTHD analysed the Illumina sequencing, prepared figures, and tables, reviewed the draft, and approved the final draft. BB analysed the Illumina sequencing, reviewed the draft, and approved the final draft. WR designed the experiments. AB reviewed draft and approved the final draft. RS helped design experiments, helped to analyse the data, co-authored or reviewed drafts of the paper, and approved the final draft. All authors contributed to the article and approved the submitted version.

## Funding

This work is supported by a BE-Basic Foundation-FES grant No. 0905 from the Ministry of Economic Affairs, The Netherlands, and a grant (code 826/QD-BKHCN) from the Ministry of Science and Technology in Vietnam (MOST).

## Conflict of interest

AB was employed by BioDetection Systems.

The remaining authors declare that the research was conducted in the absence of any commercial or financial relationships that could be construed as a potential conflict of interest.

## Publisher’s note

All claims expressed in this article are solely those of the authors and do not necessarily represent those of their affiliated organizations, or those of the publisher, the editors and the reviewers. Any product that may be evaluated in this article, or claim that may be made by its manufacturer, is not guaranteed or endorsed by the publisher.
