# Supplementary material for: Correlating biodegradation kinetics of 2,3,7,8-tetrachlorodibenzo-p-dioxin to the dynamics of microbial communities originating from soil in Vietnam contaminated with herbicides and dioxins
Source: Front Microbiol. 2022 Aug 11;13:923432. doi: 10.3389/fmicb.2022.923432 (PMC9404497; doi:10.3389/fmicb.2022.923432)
Supplement: Supplementary file 1 [file Data_Sheet_1.docx]

**Supplementary**

Table S1. Primers used for amplicon sequencing

Forward primers used for amplicon sequencing

| **Primer Name** | **Adapter** | **i5** | **Pad-forward** | **Link-V3f** | **V3-16Sf** |  |  |
| --- | --- | --- | --- | --- | --- | --- | --- |
| V3.SA501 | AATGATACGGCGACCACCGAGATCTACAC | ATCGTACG | TATGGTAATT | GG | CCTACGGGNGGCWGCAG | **V3.SA501F** | AATGATACGGCGACCACCGAGATCTACACATCGTACGTATGGTAATTGGCCTACGGGNGGCWGCAG |
| V3.SA502 | AATGATACGGCGACCACCGAGATCTACAC | ACTATCTG | TATGGTAATT | GG | CCTACGGGNGGCWGCAG | **V3.SA502F** | AATGATACGGCGACCACCGAGATCTACACACTATCTGTATGGTAATTGGCCTACGGGNGGCWGCAG |
| V3.SA503 | AATGATACGGCGACCACCGAGATCTACAC | TAGCGAGT | TATGGTAATT | GG | CCTACGGGNGGCWGCAG | **V3.SA503F** | AATGATACGGCGACCACCGAGATCTACACTAGCGAGTTATGGTAATTGGCCTACGGGNGGCWGCAG |
| V3.SA504 | AATGATACGGCGACCACCGAGATCTACAC | CTGCGTGT | TATGGTAATT | GG | CCTACGGGNGGCWGCAG | **V3.SA504F** | AATGATACGGCGACCACCGAGATCTACACCTGCGTGTTATGGTAATTGGCCTACGGGNGGCWGCAG |
| V3.SA505 | AATGATACGGCGACCACCGAGATCTACAC | TCATCGAG | TATGGTAATT | GG | CCTACGGGNGGCWGCAG | **V3.SA505F** | AATGATACGGCGACCACCGAGATCTACACTCATCGAGTATGGTAATTGGCCTACGGGNGGCWGCAG |
| V3.SA506 | AATGATACGGCGACCACCGAGATCTACAC | CGTGAGTG | TATGGTAATT | GG | CCTACGGGNGGCWGCAG | **V3.SA506F** | AATGATACGGCGACCACCGAGATCTACACCGTGAGTGTATGGTAATTGGCCTACGGGNGGCWGCAG |
| V3.SA507 | AATGATACGGCGACCACCGAGATCTACAC | GGATATCT | TATGGTAATT | GG | CCTACGGGNGGCWGCAG | **V3.SA507F** | AATGATACGGCGACCACCGAGATCTACACGGATATCTTATGGTAATTGGCCTACGGGNGGCWGCAG |
| V3.SA508 | AATGATACGGCGACCACCGAGATCTACAC | GACACCGT | TATGGTAATT | GG | CCTACGGGNGGCWGCAG | **V3.SA508F** | AATGATACGGCGACCACCGAGATCTACACGACACCGTTATGGTAATTGGCCTACGGGNGGCWGCAG |

Reverse primers used for amplicon sequencing

| **Primer Name** | **Adapter** | **i7** | **i7_reverse_complement** | **Pad-reverse** | **Link-V4r** | **V4-16Sr** |  |  |
| --- | --- | --- | --- | --- | --- | --- | --- | --- |
| v4.SA701 | CAAGCAGAAGACGGCATACGAGAT | AACTCTCG | CGAGAGTT | AGTCAGTCAG | CC | GGACTACHVGGGTWTCTAAT | **v4.SA701R** | CAAGCAGAAGACGGCATACGAGATAACTCTCGAGTCAGTCAGCCGGACTACHVGGGTWTCTAAT |
| v4.SA702 | CAAGCAGAAGACGGCATACGAGAT | ACTATGTC | GACATAGT | AGTCAGTCAG | CC | GGACTACHVGGGTWTCTAAT | **v4.SA702R** | CAAGCAGAAGACGGCATACGAGATACTATGTCAGTCAGTCAGCCGGACTACHVGGGTWTCTAAT |
| v4.SA703 | CAAGCAGAAGACGGCATACGAGAT | AGTAGCGT | ACGCTACT | AGTCAGTCAG | CC | GGACTACHVGGGTWTCTAAT | **v4.SA703R** | CAAGCAGAAGACGGCATACGAGATAGTAGCGTAGTCAGTCAGCCGGACTACHVGGGTWTCTAAT |
| v4.SA704 | CAAGCAGAAGACGGCATACGAGAT | CAGTGAGT | ACTCACTG | AGTCAGTCAG | CC | GGACTACHVGGGTWTCTAAT | **v4.SA704R** | CAAGCAGAAGACGGCATACGAGATCAGTGAGTAGTCAGTCAGCCGGACTACHVGGGTWTCTAAT |
| v4.SA705 | CAAGCAGAAGACGGCATACGAGAT | CGTACTCA | TGAGTACG | AGTCAGTCAG | CC | GGACTACHVGGGTWTCTAAT | **v4.SA705R** | CAAGCAGAAGACGGCATACGAGATCGTACTCAAGTCAGTCAGCCGGACTACHVGGGTWTCTAAT |
| v4.SA706 | CAAGCAGAAGACGGCATACGAGAT | CTACGCAG | CTGCGTAG | AGTCAGTCAG | CC | GGACTACHVGGGTWTCTAAT | **v4.SA706R** | CAAGCAGAAGACGGCATACGAGATCTACGCAGAGTCAGTCAGCCGGACTACHVGGGTWTCTAAT |
| v4.SA707 | CAAGCAGAAGACGGCATACGAGAT | GGAGACTA | TAGTCTCC | AGTCAGTCAG | CC | GGACTACHVGGGTWTCTAAT | **v4.SA707R** | CAAGCAGAAGACGGCATACGAGATGGAGACTAAGTCAGTCAGCCGGACTACHVGGGTWTCTAAT |
| v4.SA708 | CAAGCAGAAGACGGCATACGAGAT | GTCGCTCG | CGAGCGAC | AGTCAGTCAG | CC | GGACTACHVGGGTWTCTAAT | **v4.SA708R** | CAAGCAGAAGACGGCATACGAGATGTCGCTCGAGTCAGTCAGCCGGACTACHVGGGTWTCTAAT |
| v4.SA709 | CAAGCAGAAGACGGCATACGAGAT | GTCGTAGT | ACTACGAC | AGTCAGTCAG | CC | GGACTACHVGGGTWTCTAAT | **v4.SA709R** | CAAGCAGAAGACGGCATACGAGATGTCGTAGTAGTCAGTCAGCCGGACTACHVGGGTWTCTAAT |
| v4.SA710 | CAAGCAGAAGACGGCATACGAGAT | TAGCAGAC | GTCTGCTA | AGTCAGTCAG | CC | GGACTACHVGGGTWTCTAAT | **v4.SA710R** | CAAGCAGAAGACGGCATACGAGATTAGCAGACAGTCAGTCAGCCGGACTACHVGGGTWTCTAAT |
| v4.SA711 | CAAGCAGAAGACGGCATACGAGAT | TCATAGAC | GTCTATGA | AGTCAGTCAG | CC | GGACTACHVGGGTWTCTAAT | **v4.SA711R** | CAAGCAGAAGACGGCATACGAGATTCATAGACAGTCAGTCAGCCGGACTACHVGGGTWTCTAAT |
| v4.SA712 | CAAGCAGAAGACGGCATACGAGAT | TCGCTATA | TATAGCGA | AGTCAGTCAG | CC | GGACTACHVGGGTWTCTAAT | **v4.SA712R** | CAAGCAGAAGACGGCATACGAGATTCGCTATAAGTCAGTCAGCCGGACTACHVGGGTWTCTAAT |
| v4.SB701 | CAAGCAGAAGACGGCATACGAGAT | AAGTCGAG | CTCGACTT | AGTCAGTCAG | CC | GGACTACHVGGGTWTCTAAT | **v4.SB701R** | CAAGCAGAAGACGGCATACGAGATAAGTCGAGAGTCAGTCAGCCGGACTACHVGGGTWTCTAAT |
| v4.SB702 | CAAGCAGAAGACGGCATACGAGAT | ATACTTCG | CGAAGTAT | AGTCAGTCAG | CC | GGACTACHVGGGTWTCTAAT | **v4.SB702R** | CAAGCAGAAGACGGCATACGAGATATACTTCGAGTCAGTCAGCCGGACTACHVGGGTWTCTAAT |
| v4.SB703 | CAAGCAGAAGACGGCATACGAGAT | AGCTGCTA | TAGCAGCT | AGTCAGTCAG | CC | GGACTACHVGGGTWTCTAAT | **v4.SB703R** | CAAGCAGAAGACGGCATACGAGATAGCTGCTAAGTCAGTCAGCCGGACTACHVGGGTWTCTAAT |
| v4.SB704 | CAAGCAGAAGACGGCATACGAGAT | CATAGAGA | TCTCTATG | AGTCAGTCAG | CC | GGACTACHVGGGTWTCTAAT | **v4.SB704R** | CAAGCAGAAGACGGCATACGAGATCATAGAGAAGTCAGTCAGCCGGACTACHVGGGTWTCTAAT |

Sequencing primers

| **V3F_seqprim F** | TATGGTAATTGGCCTACGGGNGGCWGCAG |  |
| --- | --- | --- |
| **V4F_seqprim R** | AGTCAGTCAGCCGGACTACHVGGGTWTCTAAT | |
| **V4P7_index** | ATTAGAWACCCBDGTAGTCCGGCTGACTGACT | |

Table S2. PCR program used during the amplicon sequencing

| 1x | 98°C | 30 sec |
| --- | --- | --- |
|  | 98°C | 10 sec |
| 30x | 55°C | 30 sec |
|  | 72°C | 30 sec |
| 1x | 72°C | 5 min |

Table S3. The number of reads and the good's coverage values each samples

| Carbon source | Repeat | Day of incubation | Number of read | Goods_coverage |
| --- | --- | --- | --- | --- |
| 3mM AA | 1 | 0d | 27615 | 99.97 |
|  | 2 | 0d | 13333 | 99.94 |
|  | 1 | 10d | 25352 | 99.96 |
|  | 2 | 10d | 12579 | 99.97 |
|  | 1 | 28d | 16478 | 99.99 |
|  | 2 | 28d | 17098 | 99.98 |
|  | 1 | 45d | 42195 | 99.96 |
|  | 2 | 45d | 38023 | 100.00 |
|  | 1 | 60d | 58888 | 99.96 |
|  | 2 | 60d | 27398 | 99.96 |
|  | 1 | 90d | 46738 | 99.93 |
|  | 2 | 90d | 28832 | 99.96 |
|  | 1 | 120d | 78431 | 99.96 |
|  | 2 | 120d | 97373 | 99.95 |
| No additional carbon | 1 | 0d | 14785 | 99.94 |
|  | 2 | 0d | 21797 | 99.93 |
|  | 1 | 10d | 46658 | 99.93 |
|  | 2 | 10d | 26968 | 99.96 |
|  | 1 | 28d | 34800 | 99.95 |
|  | 2 | 28d | 69392 | 99.90 |
|  | 1 | 45d | 37647 | 99.93 |
|  | 2 | 45d | 25848 | 99.93 |
|  | 1 | 60d | 17490 | 99.93 |
|  | 2 | 60d | 34628 | 99.93 |
|  | 1 | 90d | 25823 | 99.91 |
|  | 2 | 90d | 16150 | 99.93 |
|  | 1 | 120d | 90332 | 99.94 |
|  | 2 | 120d | 82402 | 99.96 |
| 5 mM Sa | 1 | 0d | 16199 | 99.93 |
|  | 2 | 0d | 14977 | 99.92 |
|  | 1 | 10d | 15930 | 99.95 |
|  | 2 | 10d | 29801 | 99.96 |
|  | 1 | 28d | 33319 | 99.94 |
|  | 2 | 28d | 33125 | 99.93 |
|  | 1 | 45d | 20586 | 99.94 |
|  | 2 | 45d | 35100 | 99.96 |
|  | 1 | 60d | 56216 | 99.94 |
|  | 2 | 60d | 47573 | 99.95 |
|  | 1 | 90d | 23772 | 99.91 |
|  | 2 | 90d | 44464 | 99.97 |
|  | 1 | 120d | 111300 | 99.94 |
|  | 2 | 120d | 90816 | 99.94 |
| 12 mM Ace+ 9 mM Lac | 1 | 0d | 13658 | 99.94 |
|  | 2 | 0d | 16196 | 99.97 |
|  | 1 | 10d | 24053 | 99.95 |
|  | 2 | 10d | 26314 | 99.97 |
|  | 1 | 28d | 23342 | 99.95 |
|  | 2 | 28d | 47482 | 99.96 |
|  | 1 | 45d | 31214 | 99.92 |
|  | 2 | 45d | 32516 | 99.93 |
|  | 2 | 60d | 38127 | 99.93 |
|  | 1 | 60d | 12115 | 99.88 |
|  | 1 | 90d | 28790 | 99.91 |
|  | 2 | 90d | 33471 | 99.96 |
|  | 1 | 120d | 92221 | 99.97 |
|  | 2 | 120d | 93575 | 99.94 |
| 1mM V | 1 | 0d | 30514 | 99.97 |
|  | 2 | 0d | 17163 | 99.96 |
|  | 1 | 10d | 24390 | 99.95 |
|  | 2 | 10d | 28249 | 99.94 |
|  | 1 | 28d | 23057 | 99.97 |
|  | 2 | 28d | 23793 | 99.98 |
|  | 1 | 45d | 30261 | 99.97 |
|  | 2 | 45d | 15430 | 99.92 |
|  | 1 | 60d | 22999 | 99.95 |
|  | 2 | 60d | 43675 | 99.97 |
|  | 1 | 90d | 23934 | 99.94 |
|  | 2 | 90d | 27478 | 99.95 |
|  | 1 | 120d | 85541 | 99.93 |
|  | 2 | 120d | 98726 | 99.95 |
| 5mM V | 1 | 0d | 14681 | 99.93 |
|  | 2 | 0d | 17170 | 99.95 |
|  | 1 | 10d | 28202 | 99.96 |
|  | 2 | 10d | 23991 | 99.97 |
|  | 1 | 28d | 16137 | 99.93 |
|  | 2 | 28d | 18266 | 99.94 |
|  | 1 | 45d | 22543 | 99.93 |
|  | 2 | 45d | 22680 | 99.95 |
|  | 1 | 60d | 22317 | 99.95 |
|  | 2 | 60d | 44928 | 99.94 |
|  | 1 | 90d | 49711 | 99.95 |
|  | 2 | 90d | 19273 | 99.92 |
|  | 1 | 120d | 91334 | 99.95 |
|  | 2 | 120d | 90578 | 99.95 |

Table S4. Average dioxin toxicity of T1 soil at the start of bioremediation.

| **Ordinal numbers** | **Compound** | **TEF (WHO 2005)** | **ngTEQ/kg** |
| --- | --- | --- | --- |
| 1 | 2,3,7,8-TCDD | 1,00 | 21.476 |
| 2 | 1,2,3,7,8-PeCDD | 1,00 | 75,4 |
| 3 | 1,2,3,4,7,8-HxCDD | 0,10 | 7,88 |
| 4 | 1,2,3,6,7,8-HxCDD | 0,10 | 76,2 |
| 5 | 1,2,3,7,8,9-HxCDD | 0,10 | 39,0 |
| 6 | 1,2,3,4,6,7,8-HpCDD | 0,01 | 240 |
| 7 | OCDD | 0,00 | 805 |
| 8 | 2,3,7,8-TCDF | 0,10 | 332 |
| 9 | 1,2,3,7,8-PeCDF | 0,03 | 6,00 |
| 10 | 2,3,4,7,8-PeCDF | 0,30 | 8,63 |
| 11 | 1,2,3,4,7,8-HxCDF | 0,10 | 8,63 |
| 12 | 1,2,3,6,7,8-HxCDF | 0,10 | 4,88 |
| 13 | 2,3,4,6,7,8-HxCDF | 0,10 | 2,63 |
| 14 | 1,2,3,7,8,9-HxCDF | 0,10 | 1,50 |
| 15 | 1,2,3,4,6,7,8-HpCDF | 0,01 | 39,4 |
| 16 | 1,2,3,4,7,8,9-HpCDF | 0,01 | 3,00 |
| 17 | OCDF | 0,00 | 61,5 |
| **Total** | **PCDD/F – TEQ (WHO 2005)** |  | **21.605** |

**Figures**

**
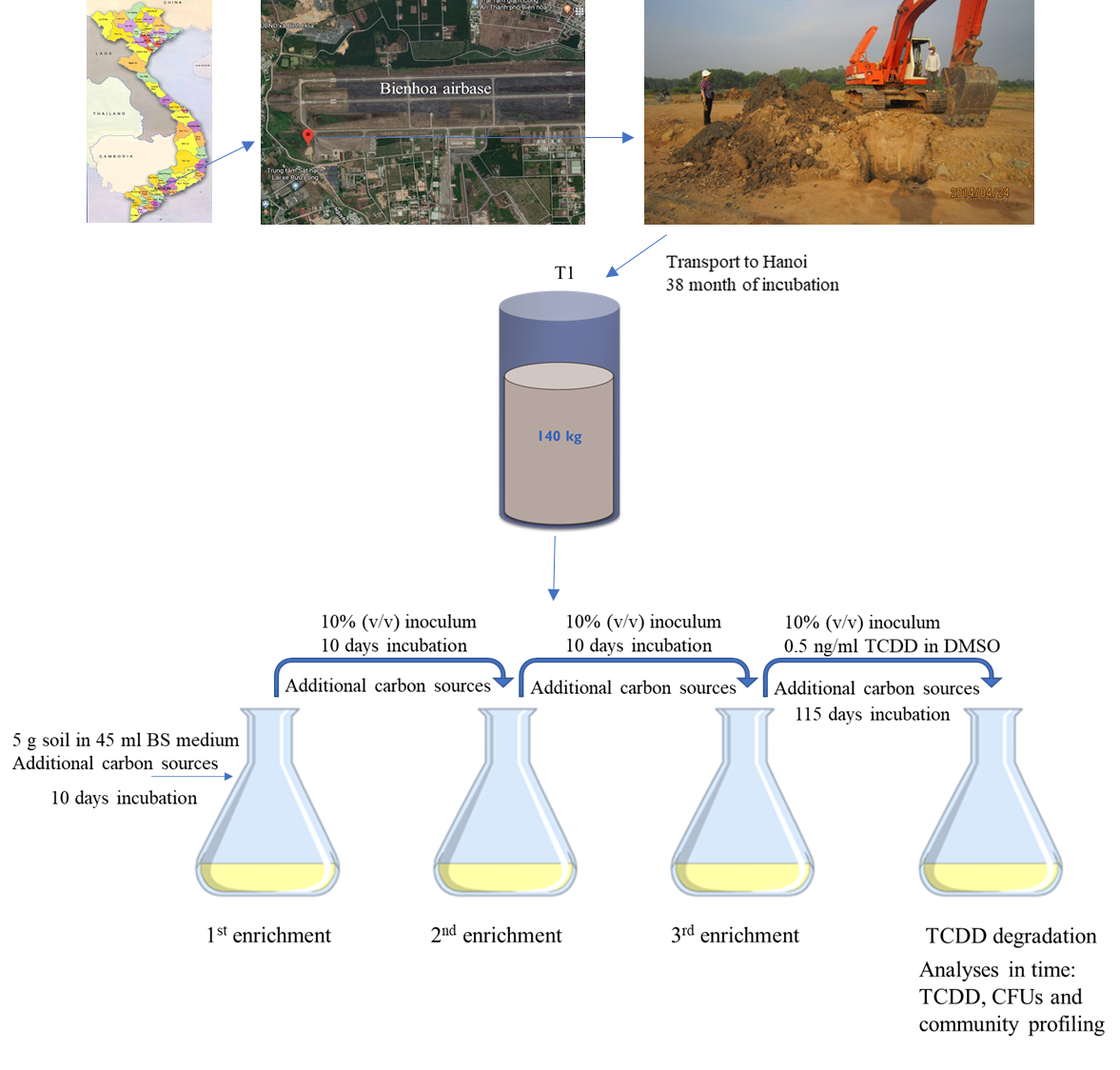
**

Figure S1. Map of Bien Hoa airbase with the position of soil samples contaminated with herbicides that were investigated during this study (top left and middle), and details of the sampling (top right), and a cartoon of containers T1 showing the amounts in kg and sampling times (bottom). Credits Google Earth. Herbicide and dioxin-contaminated soil was collected from Bien Hoa airbase (10°58’14.3” N 123 106°48’19.3” E), Dong Nai Province, Vietnam. Additional carbon sources were vanillin, a mixture of acetate and lactate, aromatic amino acids and salicylate. Contaminated soil was enriched in BS medium supplied with additional carbon sources after 10 days of cultivation (1^st^ enrichment). Then 10% (v/v) of these cultures were transferred into fresh BS medium with the addition of the same carbon sources during 10 days of cultivation (2^nd^ enrichment). The 3^rd^ enrichment steps were done the same way as described for the 2^nd^ enrichment. After a 10-day period, 10% (v/v) of the 3^rd^ enrichments were transferred into fresh BS medium with the same carbon sources described above and with the addition of 2,3,7,8-TCDD at final concentrations of 0.5 ng 2,3,7,8-TCDD TEQ/ml. These cultures were incubated during 115 days.


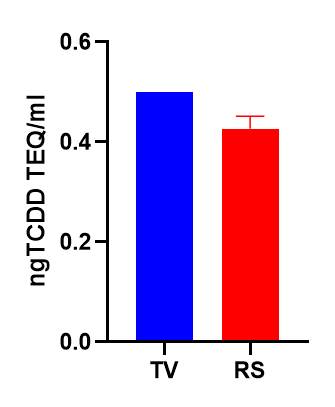


Figure S2. 2,3,7,8-TCDD concentration in random sampling by DR CALUX assay. The error bar at each data point represents the standard error of three independent experiment. TV: Theoretical value, RS: Random sampling-based analysis values.

Figure S3: Number of CFUs from cultures in BS medium during incubations on different carbon and energy sources in addition to 7 mM dimethyl sulfoxide (DMSO). No additional carbon, 1 mM vanillin (1 mM V), 5 mM vanillin (5 mM V), 5 mM sodium salicylate (5 mM Sa) and a mixture of 12 mM sodium acetate and 9 mM sodium lactate (12 mM Ace+ 9 mM Lac), 3 mM aromatic amino acids (3 mM AA). The error bar at each data point represents the standard deviation of two independent experiments.


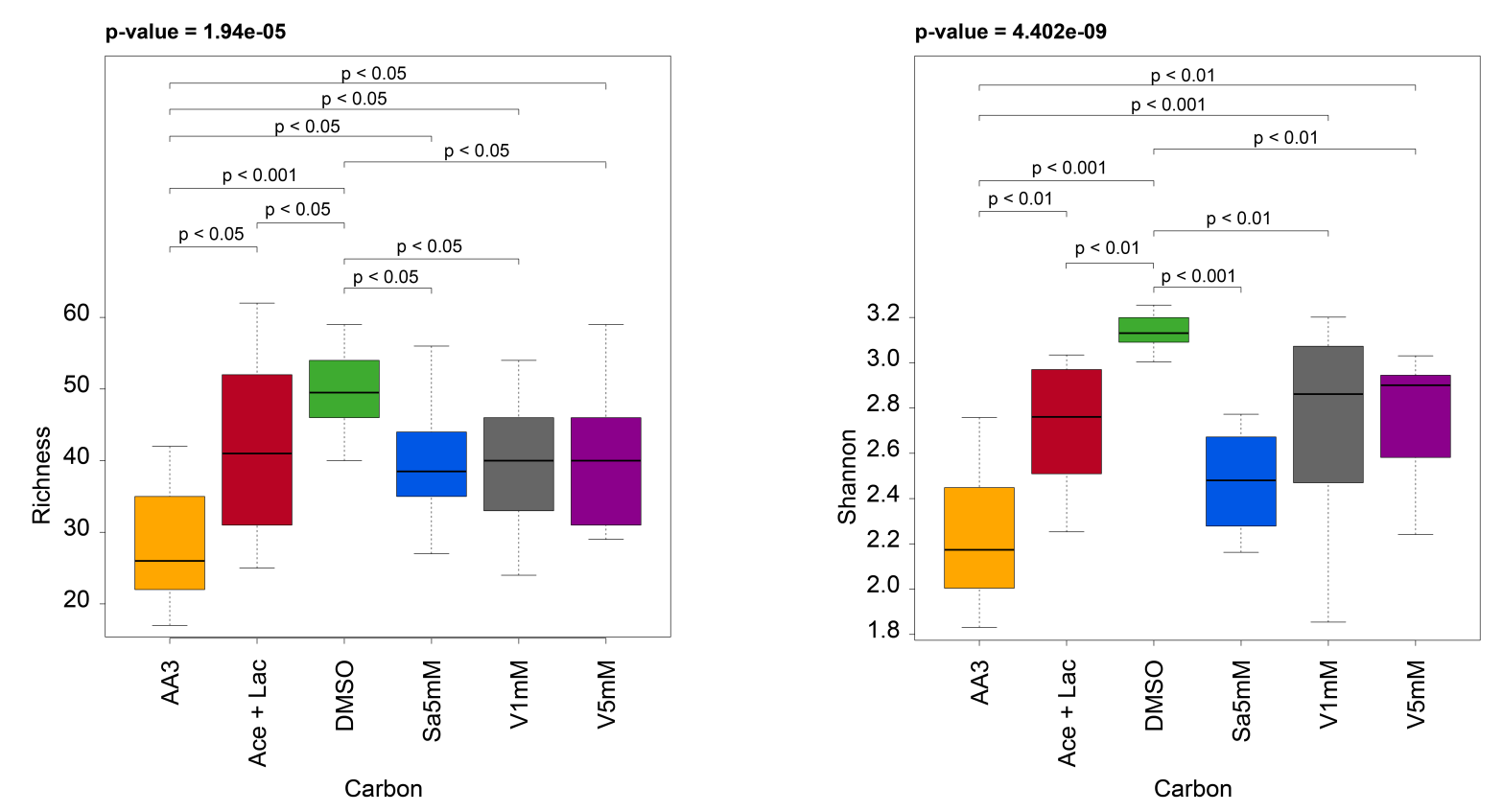


Figure S4. Alpha diversity index of bacterial communities from cultures in BS medium during growth on different carbon and energy sources in addition to 7 mM dimethyl sulfoxide (DMSO). AA3, 3 mM aromatic amino acids; Ace + Lac, a mixture of 12 mM sodium acetate and 9 mM sodium lactate; DMSO, no additional carbon; Sa5mM, 5mM sodium salicylate; V1mM, 1 mM vanillin; V5mM, 5 mM vanillin. Chao1 richness indices are shown at the left panel and the Shannon indices at the right panel.


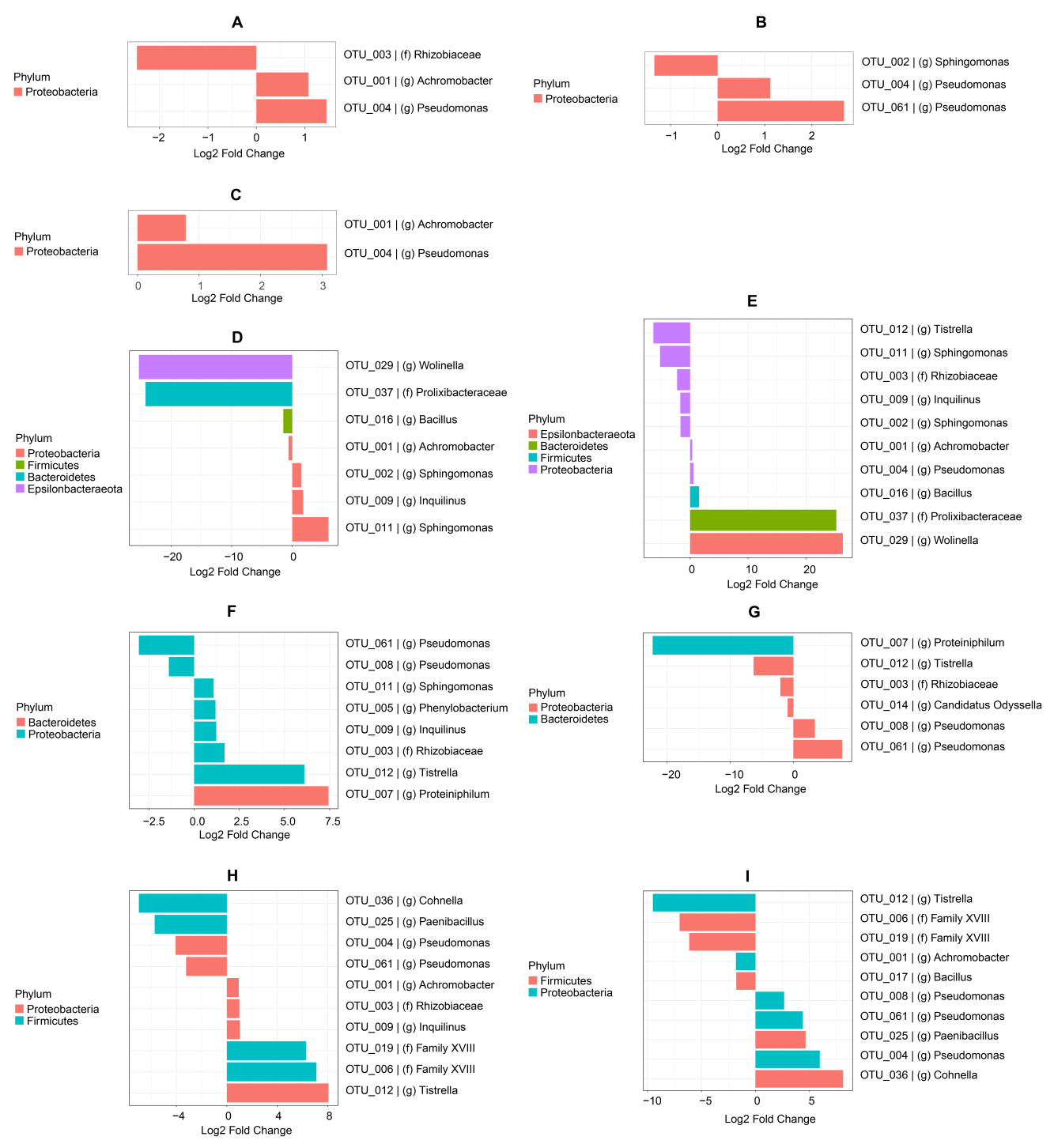


Figure S5. Differential analysis representing OTUs whose relative abundance changed significantly (p < 0.05) after 60 (D, F, H) and 115 days (A, B, C, E, G, I) of incubation compared to day 28 in cultures with BS medium growing on different carbon and energy sources in addition to 7 mM dimethyl sulfoxide (DMSO). The different sources are a mixture of tryptophan 1 mM, tyrosine 1 mM and phenylalanine 1 mM (A), no additional carbon and energy sources (B), 5 mM sodium salicylate (C), a mixture of 12 mM sodium acetate and 9 mM sodium lactate (D and E), 1 mM vanillin (F and G) and 5 mM vanillin (H and I). Coloured bars represent the phylum of the presented OTUs.
